# Supplementary material for: BGM-YOLO: An accurate and efficient detector for detecting plant disease
Source: PLoS One. 2025 May 28;20(5):e0322750. doi: 10.1371/journal.pone.0322750 (PMC12119003; doi:10.1371/journal.pone.0322750)
Supplement: Attachment — Submitted filename: Response to Reviewers.docx [file pone.0322750.s002.docx]

# Responses to Reviewers’ Comments

**Reviewer 1**

| **#** | **Comments** | **Responses** |
| --- | --- | --- |
| 1 | The author should show how the accuracy of network increases as more images are provided for training and compare that to the base line models. It would be nice to know how many images are needed for the proposed method to be more accurate than models it is compared to. | Revised.  Thank you for your valuable comments. In this study, our model was trained from scratch without using any pre-trained weights. This approach ensures that the network learns domain-specific features directly for plant disease detection, rather than relying on pre-trained models from other datasets. By doing so, the model can better adapt to the unique patterns of plant disease data, enhancing its performance in the target domain. To address this concern, we have added a clarification in Section 3.2 of the paper to ensure that readers fully understand our training strategy. Once again, we appreciate your review and suggestions. |
| 2 | The author should show how the accuracy of network increases as more images are provided for training and compare that to the base line models. It would be nice to know how many images are needed for the proposed method to be more accurate than models it is compared to. | Revised.  Thank you for your valuable suggestion. We have addressed this concern by adding a comprehensive analysis in Section 3.6 of our revised manuscript. We conducted experiments training our model with varying amounts of data (20%, 40%, 60%, 80%, and 100% of the training dataset) and evaluated performance on the test set. Figure 9 illustrates how detection accuracy (mAP50 and mAP50:95) increases with training data volume. |
| 3 | Each plant species should have a healthy class in addition to diseased classes. If you have diseases that are specific to some plant species in your data, then the model may just be predicting what plant species it is. | Revised.  Thank you for raising this important methodological consideration. We have carefully addressed this concern in our study design.  As detailed in Section 3.1 of our manuscript, we have included healthy class data for each plant species in our dataset—for beans, strawberries, and tomatoes. This approach ensures that our model is learning to distinguish between healthy and diseased states rather than simply identifying plant species.  In Section 3.3, we present the performance metrics of our model, which demonstrate its ability to accurately classify both healthy and diseased conditions across all three plant species. The inclusion of healthy classes for each plant type allows our model to establish proper baselines for normal plant appearance, which is essential for reliable disease detection.  By incorporating these healthy classes in our training data, we have effectively controlled for the potential confounding factor you've identified. Our model is therefore trained to recognize disease-specific features rather than merely differentiating between plant species.  The performance results in Section 3.3 further validate this approach, showing that the model successfully identifies both healthy specimens and various disease states within the same plant species, confirming that the classification is based on disease characteristics rather than species-specific traits. |
| 4 | It is unclear if the model developed in the manuscript has a higher accuracy for identifying the bounding boxes. Something like the Jaccard index should be used to quantify how accurate the bounding boxes are from the models. | Revised.  Thank you for your suggestion regarding the evaluation of bounding box accuracy in our model.  In our manuscript, we have indeed addressed this concern in Section 3.3, where we provide a detailed explanation of the mAP50 and mAP50:95 metrics. These standard object detection evaluation metrics inherently quantify bounding box accuracy through Intersection over Union (IoU) measurements.  The mAP50 metric evaluates detection accuracy with an IoU threshold of 0.5, while mAP50:95 provides a more comprehensive assessment by averaging performance across multiple IoU thresholds (from 0.5 to 0.95 in 0.05 increments). This approach effectively measures how precisely our model localizes objects across varying degrees of overlap requirements.  Section 3.3 elaborates on how these metrics specifically reflect the spatial accuracy of our bounding box predictions and demonstrates our model's capability to precisely localize plant disease regions. The higher mAP50:95 values achieved by our model, as detailed in this section, indicate superior bounding box precision compared to baseline approaches.  We appreciate your recommendation regarding the Jaccard index, which is mathematically equivalent to IoU. The IoU measurements underlying our reported mAP metrics serve the same purpose of quantifying the spatial overlap between predicted and ground truth bounding boxes. |
| 5 | The results should be split into each plant species with multiple diseases and the healthy classes. This would help prospective users of the model understand how it works across species. | Revised.  Thank you for your valuable comment regarding species-specific performance analysis. We have addressed this concern by expanding Section 3.3 of our manuscript to include detailed performance metrics across different plant species and their associated disease classes.  In our revised manuscript, we have incorporated comprehensive class-wise performance metrics including Precision (P), Recall (R), mAP50, and mAP50:95 for each plant species and their corresponding disease conditions. Table 1 now presents these detailed metrics, demonstrating the model's performance variation across different plant categories.  This species-specific performance breakdown provides prospective users with greater insight into the model's strengths and limitations across different plant types and disease conditions, allowing for more informed implementation decisions based on their specific agricultural monitoring needs. |
| 6 | Confusion matrices of the test data predictions should be added so the readers understand the nature of the errors the model makes. | Revised.  Thank you for your suggestion regarding the inclusion of confusion matrices. We have addressed this recommendation by incorporating a comprehensive confusion matrix analysis in Section 3.3 of our revised manuscript.  Figure 8 now presents the confusion matrix for our test dataset, providing a clear visualization of the model's prediction patterns across all disease categories. This addition offers readers valuable insight into both the strengths and limitations of our detection system.  The confusion matrix reveals strong diagonal values for most classes, confirming the high accuracy rates reflected in our performance metrics. However, it also illuminates specific patterns of misclassification that warrant attention. Notably, we observed confusion between visually similar disease categories, such as Beans_Angular_LeafSpot and Beans_Rust, as well as between Strawberry_Powdery_Mildew_Leaf and Strawberry_Powdery_Mildew_Fruit.  These patterns of misclassification highlight an important challenge in plant disease detection: diseases with similar visual manifestations can be difficult to distinguish, even for advanced deep learning models. This finding provides valuable direction for future improvements, suggesting that enhanced feature extraction techniques or additional contextual information may be necessary to further refine classification accuracy between visually similar disease categories.  The inclusion of this confusion matrix analysis strengthens our manuscript by providing readers with a more nuanced understanding of model performance and the specific nature of detection errors, which is essential for potential users considering implementation in agricultural monitoring systems. |
| 7 | Do table 1 & 2 really need to be tables? You could just state them in materials and methods. | Revised.  Thank you for your feedback regarding Tables 1 and 2. We agree with your suggestion and have revised our manuscript accordingly, replacing the tabular presentation of experimental environment and hyperparameter settings with a more concise textual description in Section 3.2.  The experimental setup and parameters are now integrated into the main text。  This narrative format improves readability while maintaining all essential information about our experimental configuration. The description provides a comprehensive overview of the hardware specifications, software environment, and training parameters in a more flowing, accessible manner than the previous tabular presentation.  We believe this revision enhances the overall structure of the manuscript while ensuring that readers still have access to all necessary methodological details for replication purposes. |
| 8 | Figure 3 is too small to see what is going on in each image. | Revised.  We acknowledge your concern regarding the size of Figure 3 and appreciate your feedback. In our revised manuscript, we have significantly enlarged Figure to improve visibility and readability of the image details. |
| 9 | Could the new model better quantify the severity of the disease? | Revised.  Thank you for raising this important question about disease severity quantification. While our current research primarily focuses on disease detection and identification rather than severity assessment, your question addresses a valuable potential application of our model.  Our approach does not explicitly incorporate disease severity measurement as a primary research objective. The model has been designed and trained to accurately detect and classify various plant diseases rather than to quantify their progression or impact levels.  That said, we acknowledge that the confidence scores associated with each bounding box prediction could potentially serve as a proxy indicator for disease severity. Higher confidence scores might correlate with more visually prominent disease symptoms, which could indirectly relate to severity. However, this relationship would require thorough validation before being considered reliable for practical applications.  For future research, we recognize the significant value in extending our model to incorporate severity assessment capabilities. This would likely require:   1. Training data specifically annotated with severity levels 2. Modified model architecture to output severity scores 3. Validation against expert assessment of disease progression   Such an enhancement would significantly increase the practical utility of our system for agricultural management, enabling not just detection but also prioritization of intervention based on disease severity.  We appreciate your suggestion and will consider it as a promising direction for future development of our plant disease detection framework. |
| 10 | Could a well-known pretrained model such as resnet50 to be added to part of the classifier of the new model? | Revised.  Thank you for your question regarding the integration of pre-trained models such as ResNet50 into our classifier architecture.  Our decision not to incorporate additional classifiers like ResNet50 was deliberate and aligned with our primary design objectives. The YOLO architecture already provides robust classification capabilities within its detection framework, making additional classifiers redundant for our specific application.  There were two key considerations that informed this decision:  First, model efficiency was a critical priority in our research. Adding a pre-trained ResNet50 would significantly increase the model's computational requirements, parameter count, and inference time. This would undermine our goal of developing a lightweight solution suitable for deployment in resource-constrained agricultural environments.  Second, our experiments demonstrated that the native classification capabilities of the YOLO framework were sufficient for accurately identifying the plant diseases in our dataset. The performance metrics detailed confirm that our streamlined approach achieves high accuracy across most disease categories without requiring supplementary classification networks.  While pre-trained models like ResNet50 can offer advantages for certain applications, our results indicate that maintaining the lightweight structure of our model provided an optimal balance between accuracy and efficiency for plant disease detection tasks. |

**Reviewer 2**

| **#** | **Comments** | **Responses** |
| --- | --- | --- |
| 1 | The numerical experiments used only one dataset, which makes the works less convincing. The authors should consider evaluating the model on more datasets to demonstrate its generalization ability. | Revised.  Thank you for your insightful comment regarding the need for multiple dataset evaluation. We have addressed this concern by adding Section 3.7 to our manuscript, which demonstrates our model's generalization capabilities across different datasets.  In this new section, we present experiments conducted using the tomato leaf disease detection dataset provided by Md Faruk Alam. This dataset includes seven disease categories (Bacterial Spot, Early Blight, Healthy, Late Blight, Leaf Mold, Target Spot, and Black Spot) and offers a valuable opportunity to test our model in a different context. Results confirm that our model maintains strong generalization capabilities even when applied to external datasets with different disease distributions. The performance improvements in specific categories demonstrate the effectiveness of our optimization methods in enhancing detection capabilities while preserving overall model stability.  This additional validation strengthens the credibility of our approach and provides readers with greater confidence in the model's applicability across diverse agricultural monitoring scenarios. |
| 2 | The authors should provide some statistical information about the dataset, e.g. the classes of the plants and/or diseases this dataset contains (and does it have a highly imbalanced distribution?); the distribution of the bounding boxes (number per image, size, etc.). | Revised.  Thank you for pointing out the need for more comprehensive dataset information. We have enhanced Section 3.1 with detailed statistical analysis of our dataset,。  This expanded description provides readers with essential quantitative insights into our dataset composition, including:   1. The precise train-validation-test split ratios 2. Plant species and disease category distribution 3. Class imbalance metrics with specific examples 4. Object density statistics (objects per image) 5. Bounding box size distribution and variability   These statistics help readers understand the complexity and characteristics of the data used for model development, providing important context for interpreting our experimental results and the model's practical applicability in agricultural disease detection scenarios. |
| 3 | Some of the figures and their titles are split into two pages. Some of the figures have low resolutions, especially Figure 8. | Revised.  Thank you for your thorough review of our manuscript and for bringing these formatting issues to our attention. We have carefully addressed all the concerns regarding the figures in our revised submission:   1. We have reformatted the document to ensure that all figures and their corresponding titles appear together on the same page, eliminating any splits between pages. 2. We have replaced all low-resolution images with high-resolution versions, with particular attention to Figure 8, which has been completely reprocessed to meet publication quality standards. 3. All figures have been reviewed for clarity and proper formatting to ensure they effectively communicate the intended information.   These improvements should enhance the overall readability and professional presentation of our manuscript. We appreciate your valuable feedback, which has helped us improve the quality of our paper. |
| 4 | Math equations are not formatted well enough (have significantly larger font size). | Revised.  Thank you for your valuable feedback regarding the math equations formatting issues in our manuscript. We appreciate your attention to detail in highlighting the significant font size discrepancies that affected the readability of the mathematical content.  We have completed all necessary modifications to address this concern:   1. We have carefully adjusted the formatting of all mathematical equations throughout the document to ensure consistent font sizing. 2. We have reviewed each equation to verify proper integration with the surrounding text, maintaining appropriate proportions and clarity. 3. We have implemented comprehensive formatting standards across all mathematical expressions to enhance visual consistency.   These improvements should significantly enhance the readability and professional presentation of the mathematical content in our work. We appreciate your guidance, which has helped us improve the overall quality of our manuscript. |
